# GSAASeqSP: A Toolset for Gene Set Association Analysis of RNA-Seq Data

**DOI:** 10.1038/srep06347

**Published:** 2014-09-12

**Authors:** Qing Xiong, Sayan Mukherjee, Terrence S. Furey

**Affiliations:** 1 Department of Computer Science and Technology, Department of Statistics, Southwest University, Chongqing 400715, China; 2 Department of Statistical Science, Department of Computer Science, and Department of Mathematics, Duke University, Durham, NC 27708, USA; 3 Department of Genetics, Department of Biology, Lineberger Comprehensive Cancer Center, and Carolina Center for Genomics and Society, The University of North Carolina at Chapel Hill, Chapel Hill, NC 27599, USA

## Abstract

RNA-Seq is quickly becoming the preferred method for comprehensively characterizing whole transcriptome activity, and the analysis of count data from RNA-Seq requires new computational tools. We developed GSAASeqSP, a novel toolset for genome-wide gene set association analysis of sequence count data. This toolset offers a variety of statistical procedures via combinations of multiple gene-level and gene set-level statistics, each having their own strengths under different sample and experimental conditions. These methods can be employed independently, or results generated from multiple or all methods can be integrated to determine more robust profiles of significantly altered biological pathways. Using simulations, we demonstrate the ability of these methods to identify association signals and to measure the strength of the association. We show that GSAASeqSP analyses of RNA-Seq data from diverse tissue samples provide meaningful insights into the biological mechanisms that differentiate these samples. GSAASeqSP is a powerful platform for investigating molecular underpinnings of complex traits and diseases arising from differential activity within the biological pathways. GSAASeqSP is available at http://gsaa.unc.edu.

Cellular processes are regulated by complex networks of functionally interacting genes. Differential activity of genes in these networks largely determines the state of the cell and cellular phenotypes. Identifying biological pathways with differential activity between phenotypically distinct samples is a powerful way to uncover molecular mechanisms underlying complex traits, diseases, and diverse cell types. Towards this end, we previously developed GSAA^1^ (Gene Set Association Analysis) that identifies differentially expressed pathways through the integration of microarray gene expression and single nucleotide polymorphism (SNP) data. In addition, a variety of alternative statistical and computational methods have been developed as well such as GSEA^2^, SAM-GS^3^, PAGE^4^, GAGE^5^, T-profiler^6^, GT^7^, AGT^8^, and GLAPA^9^. However, these programs, including GSAA, can only evaluate differential activity of pathways using real-valued data from microarrays, but not count data from RNA-seq.

RNA-Seq performs transcriptome profiling using high-throughput sequencing technologies. Compared to microarrays, RNA-Seq offers several advantages including: 1) better quantification of very high and very low expressed genes; 2) detection of all transcripts without pre-existing knowledge of their sequence or location; and 3) higher levels of reproducibility^10^. Analysis of count-based data from RNA-Seq requires the development of new methods and tools. Three existing methods have been developed for gene set analysis (GSA) of RNA-Seq data^11^,^12^,^13^,^14^: (1) SeqGSEA^11^,^12^ performs GSA using differential expression and splicing information, either independently or together, based on a weighted Kolmogorov-Smirnov (KS) statistic; (2) A GSA method proposed by Fridley et al. uses the Gamma Method with a soft truncation threshold^13^; and (3) GSVA (Gene Set Variation Analysis) calculates pathway-based variation within a sample population^14^. We found, however, that SeqGSEA is computationally intensive and only offers the single gene set-level statistic; the GSA method from Fridley et al. is not available as a public software tool; and GSVA is not designed for gene set-based differential expression analysis between two phenotypically distinct sample groups. Therefore, computational tools that assess the associations between phenotypes and differential expression of pathways for RNA-Seq data are still very much needed.

Here, we describe a novel toolset, Gene Set Association Analysis for RNA-Seq with Sample Permutation (GSAASeqSP) that efficiently performs gene set association analysis using RNA-seq count data for studies of phenotypically distinct samples. In addition to the weighted KS statistic used in SeqGSEA^11^,^12^, we adapt seven other statistics for these analyses and compare their performance within the same simulation framework demonstrating strengths and weaknesses of each statistic under differing conditions. We demonstrate the effectiveness of GSAASeqSP by using it to discover pathway differences between kidney and liver, and subtypes of breast cancer. Our toolset offers alternative options for gene set association analysis of RNA-Seq data. It will greatly assist in elucidating the molecular mechanisms underlying complex traits or human diseases. GSAASeqSP is being released as a module within our GSAA software suite that is publically available at http://gsaa.unc.edu. GSAA 1.2 now includes four functionally independent modules: GSAASeqSP, GSAASeqGP, GSAA^1^, and GSAA-SNP. These modules include different sets of analytical methods and allow for the analysis of different types of transcriptomics data and genomics data (see Supplementary Table S1 for a description of each).

## Results

### Overview of gene set association analysis in GSAASeqSP

**Figure 1:**
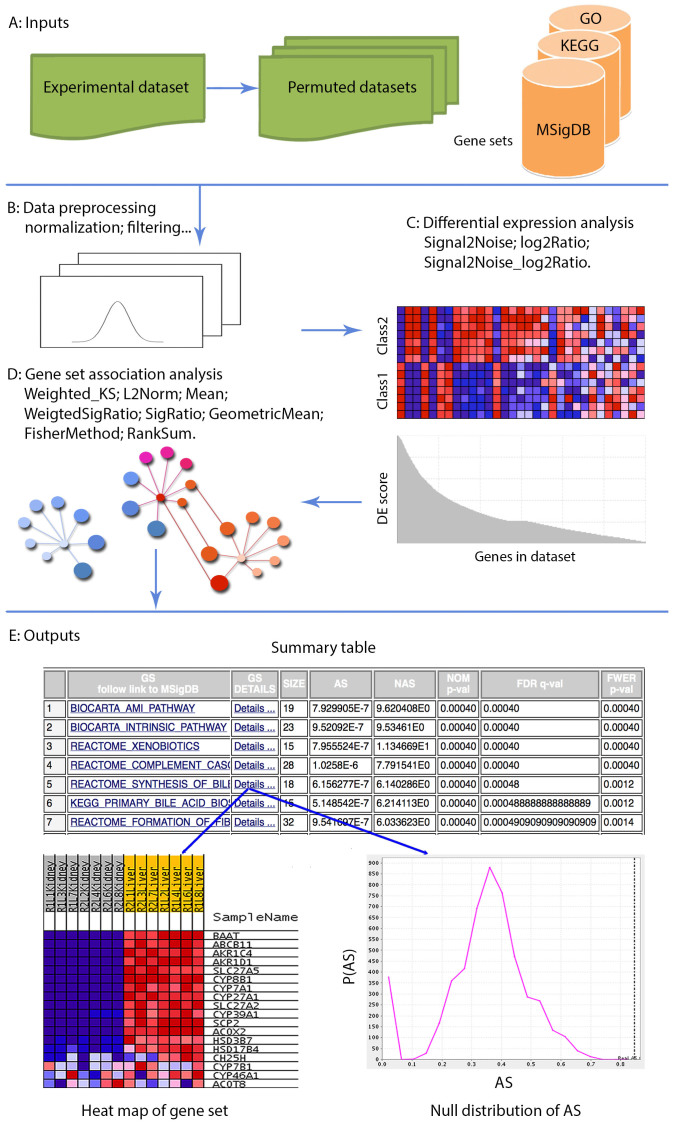
Schematic flow diagram of GSAASeqSP. (A): GSAASeqSP takes as input an experimental count dataset and *a* *priori* defined gene sets, and first generates permuted datasets based on the experimental dataset; (B): Data is normalized and extremely small and large gene sets are filtered; (C): Differential expression analysis is performed using one of: Signal2Noise, log2Ratio, and Signal2Noise_log2Ratio; (D): Gene set association analysis is performed using one of: Weighted_KS, L2Norm, Mean, WeightedSigRatio, SigRatio, GeometricMean, FisherMethod, and RankSum; (E): Outputs include 1) ranked summary gene set association table with the name of the gene set, the number of genes (SIZE), association score (AS), normalized association score (NAS), P-VALUE, FDR, and FWER; 2) a link to gene set annotation in MSigDB (where applicable); 3) a heat map of the gene expression data for each gene set; and 4) the null distribution of the AS.

GSAASeqSP takes as input RNA-seq data from multiple samples classified into two distinct phenotypic groups. Using pre-defined sets of functionally related genes, such as those in a biological pathway, GSAASeqSP identifies gene sets whose activity, as measured by gene expression, is significantly different between the two groups. To do this, GSAASeqSP employs a multi-layer statistical framework that consists of two key steps, illustrated in Figure 1: (1) differential expression analysis of individual genes between two phenotypic groups; and (2) gene set association analysis based on differential gene activity. Each step can be implemented using a variety of statistical methods. We have evaluated three gene-level statistics for differential expression analysis: Signal2Noise, log2Ratio, and Signal2Noise_log2Ratio, and ten gene set-level statistics for gene set association analysis: Weighted_KS, L2Norm, Mean, WeightedSigRatio, SigRatio, GeometricMean, TruncatedProduct, FisherMethod, MinP, and RankSum (see Methods and Supplementary Material for definitions of these statistics). Among these, one gene-level statistic (Signal2Noise_log2Ratio) and two gene set-level statistics (WeightedSigRatio, SigRatio) are proposed for the first time. The remaining statistics have been used for gene set analysis of microarray data, but the performance of these statistics, except for Weighted_KS in SeqGSEA, have not yet been evaluated using RNA-Seq data. Significance of associations is determined using sample permutation tests, and p-values, false discovery rates (FDRs), and family-wise error rates (FWERs) are reported.

### Simulation studies

A comprehensive simulation study was conducted to evaluate the performance of gene-level and gene set-level statistics under varying magnitudes and presence of signals. More specifically, we sought to determine how well each of the statistics recovered a “causal gene set” given different numbers of contributing genes in the gene set and varying effect sizes of the differentially expressed genes with respect to the association with phenotype. We designed six scenarios. In each scenario, we simulated 200 sequence count data sets each containing 1000 genes and 400 samples – 200 for each phenotype class. We simulated 100 gene sets for each data set with the first gene set being the causal gene set. The causal gene set contained sixteen genes of which a varying subset was differentially expressed. The remaining 99 gene sets were composed of a random subset of 984 non-causal genes generated from a null model. A non-causal gene may be assigned to multiple gene sets by this design. The six scenarios are distinguished by the number and magnitudes of signals embedded in the genes constituting the causal gene set:

    S1: Eight of the sixteen genes are differentially expressed, the effect size of differential expression is drawn from U[0.8, 1];

    S2: Eight of the sixteen genes are differentially expressed, the effect size of differential expression is drawn from U[1, 3];

    S3: Eight of the sixteen genes are differentially expressed, the effect size of differential expression is drawn from U[2, 4];

    S4: Twelve of the sixteen genes are differentially expressed, the effect size of differential expression is drawn from U[0.8, 1];

    S5: Twelve of the sixteen genes are differentially expressed, the effect size of differential expression is drawn from U[1, 3];

    S6: Twelve of the sixteen genes are differentially expressed, the effect size of differential expression is drawn from U[2, 4];

See Methods and Supplementary Material for more details on our simulation study design.

**Figure 2:**
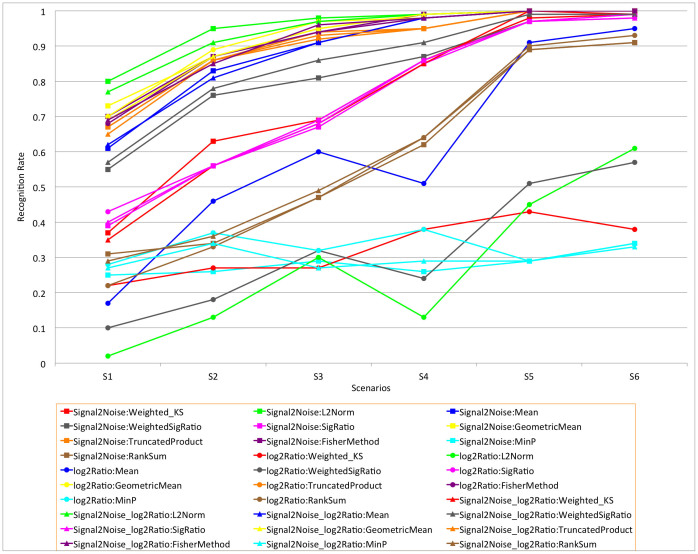
Recognition rates for all combinations of gene-level and gene set-level statistics applied to simulation scenarios 1–6.

**Figure 3:**
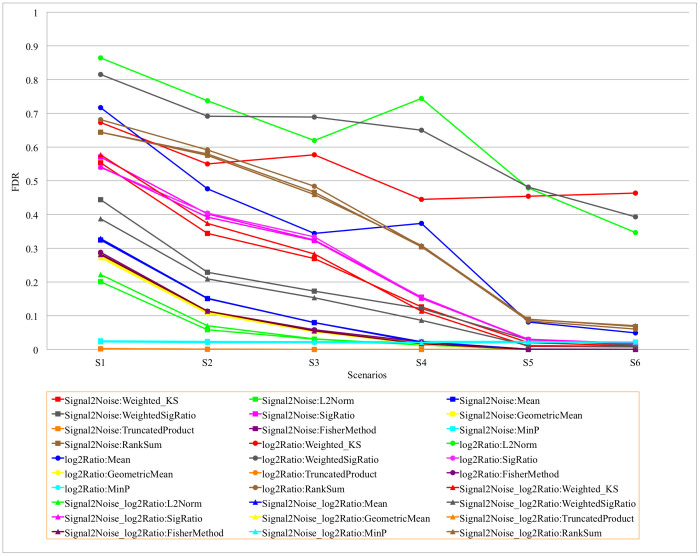
FDRs for all combinations of gene-level and gene set-level statistics applied to simulation scenarios 1–6.

We evaluated all combinations of the three gene-level statistics and ten gene set-level statistics. The results are shown in Supplementary Table S2. For each combination, we calculated the recognition rate (RR), defined as the proportion of replicates for which the causal gene set was the top-ranked gene set among the 100 gene sets, where gene sets are ranked by FDR. The average p-value, FDR and FWER for the causal gene set over 200 replicates and the power of each method in each scenario are reported as well. The p-value, FDR and FWER were calculated based on 2000 permutations of sample phenotype labels. Power was calculated as the proportion of replicates for which the p-value for the causal gene set was less than 0.05. Comparisons of RR and FDR among all gene-level and gene set-level statistical combinations are shown in Figures 2 and 3, respectively.

Overall, most combinations of gene-level statistics and gene set-level statistics can identify association signals embedded in simulated causal gene sets and distinguish the signal intensity effectively. Unsurprisingly, as the signal intensity increased, the RR and power increased while p-value, FDR, and FWER decreased. We also noticed that most combinations performed substantially better when the causal gene sets contained 12 causal genes (S4–S6) compared to those scenarios with 8 causal genes (S1–S3), as would be expected.

With respect to the recognition rate, our results show that the combination Signal2Noise (gene-level) and L2Norm (gene set-level) performed better than all other combinations (Figure 2). It achieved recognition rates of 0.80, 0.95, 0.98, 0.99, 1, and 1, respectively, for the six simulation scenarios, the highest among all combinations. Surprisingly, by including a sample based permutation procedure, several simple gene set-level statistics, such as Mean and GeometricMean, could recognize association signals effectively. Interestingly, the three combinations using MinP for the gene set association analysis performed poorly under all conditions we simulated, so we excluded these combinations from other comparisons and analyses in this simulation study. Overall, FDRs and FWERs when using TruncatedProduct as the gene set-level statistic were consistently smaller than other combinations; however the TruncatedProduct statistic showed a moderate bias towards larger gene sets in the analyses of tissue data (see Supplementary Table S3). The ranks of gene sets from this statistic were negatively correlated with gene set sizes, possibly due to the TruncatedProduct statistic only considering the significant proportion of genes in the gene set. The Signal2Noise:L2Norm gene:gene set-level statistic combination has the best overall performance based on FDR and FWER when excluding the three TruncatedProduct based combinations from the comparison.

From these simulations, we note some general characteristics of different statistics at the gene or gene set level. At the gene-level, our results show: (1) when using Weighted_KS as the gene set-level statistic, Signal2Noise performed better when there were only eight causal genes in the causal set (S1–S3) while Signal2Noise_log2Ratio was superior if there were twelve causal genes (S4–S6); (2) Signal2Noise performed the best in nearly all simulated scenarios when combined with either the L2Norm or Mean gene set-level statistic; (3) Signal2Noise_log2Ratio had the highest RR and lowest FDR and FWER in all scenarios when combined with the WeightedSigRatio gene set-level statistic; (4) All three gene-level statistics performed similarly over all simulated scenarios when the gene set-level statistic was SigRatio, GeometricMean, Truncated Product, FisherMethod, or RankSum.

The gene set-level statistics can be divided into those that take as input scores from the differential expression analysis of individual genes (Weighted_KS, L2Norm, Mean, WeightedSigRatio, SigRatio), those that take as input p-values (GeometricMean, TruncatedProduct, FisherMethod, MinP), and those that take as input ranks (RankSum). Considering the ten gene set-level statistics, our results show: (1) the L2Norm statistic performed better than all other score based statistics, and when combined with Signal2Noise or Signal2Noise_log2Ratio gene-level statistics, it had the highest RR and lowest FDR and FWER in nearly all simulated scenarios; (2) the GeometricMean statistic had the highest RR among p-value based statistics.

We implemented all of the three gene-level statistics and eight of the gene set-level statistics in our GSAASeqSP platform. The MinP and TruncatedProduct statistics were not included because MinP performed poorly in the simulations and TruncatedProduct had a size bias in the analysis of the tissue data. Our simulation study shows that different combinations perform better or worse based on characteristics of causal gene sets (proportion of differentially expressed genes, strength of association). Therefore, we do not recommend a specific combination but suggest using multiple combinations. We hypothesize that associations are more likely to be biologically meaningful if they are detected using multiple analytical methods.

### Analyses of tissue and breast cancer data

To further assess the power of GSAASeqSP to detect relevant gene sets differentiating phenotypically distinct samples, we analyzed two tissue data sets, one to explore pathway-based differences between kidney and liver tissue, and a second to identify differences between breast cancer subtypes. Our analyses of these tissue samples aimed to answer two important questions: (1) does GSAASeqSP provide biologically meaningful insights into mechanisms underlying the phenotypic distinction; and (2) were the results reproducible over multiple analytical methods. For these analyses, we used canonical pathway gene sets from the Molecular Signatures Database v4.0 (C2:CP collection, MSigDB, http://www.broadinstitute.org/gsea/msigdb/index.jsp). Pathways for which gene expression data were available for less than 15 genes or more than 100 genes in a study were filtered to avoid overly narrow or broad functional categories. This resulted in 910 and 948 canonical pathways for the kidney-liver analysis and breast cancer subtype analysis, respectively. The statistical significance of association scores for gene sets was assessed using 5000 permutations of phenotypic class labels. Signal2Noise was chosen as the gene-level statistic for differential expression analysis of individual genes since this statistic had better overall performance in our simulations. All ten gene set-level statistics were evaluated.

### Case study 1: kidney vs. liver tissue data

RNA-Seq data from kidney and liver tissue samples were generated by Marioni, et al^15^ consisting of 7 technical replicates from each tissue. The results of GSAASeqSP analyses for each of the ten gene set-level statistics paired with the Signal2Noise gene-level statistic are shown in Supplementary Tables S4–S13. In each table, pathways were sorted first by FDR, and then by the normalized association score (NAS). Results from the MinP and TruncatedProduct gene set-level statistics were excluded from all of the subsequent tissue data analyses because MinP performed poorly in simulations and TruncatedProduct was found to have a gene set size bias in these analyses. In order to find top-ranked pathways identified by multiple methods, the top 30 pathways from each of eight methods were extracted and the occurrences and ranks of each pathway were calculated, as shown in Supplementary Table S14. A “0” indicates the gene set was not ranked in the top 30 for that method. We only chose those pathways ranked in top 30 by at least four methods, and then ranked those by their average rank across those methods in which it was one of the top 30. The top ten pathways with smallest average ranks are shown in Table 1. We used the average rank for the subsequent tissue data analyses as well. While we adopted this metric for the results presented here, users should determine whether using gene sets identified as significant by all methods, by a subset of methods, or just one method provide the best results for their purposes.

We expected that biological pathways associated with kidney-specific or liver-specific functions would be identified. We found that the top 10 pathways represent several signaling cascades and metabolic processes active only or predominantly in the liver. Two pathways, BIOCARTA AMI PATHWAY (G1) and BIOCARTA INTRINSIC PATHWAY (G4), are related to the activation of the prothrombin, which is synthesized in the liver and is necessary for the coagulation of blood^16^. The coagulation cascade plays a critical role in myocardial infarction since most myocardial infarctions result from the formation of a blood clot^17^. The second-ranked pathway, REACTOME XENOBIOTICS (G2), which operates to deactivate and excrete xenobiotics, is active primarily in the liver^18^. Three pathways including REACTOME COMPLEMENT CASCADE (G3), BIOCARTA COMP PATHWAY (G5) and KEGG COMPLEMENT AND COAGULATION CASCADES (G8) represent the complement cascades and interactions between complement and coagulation systems. The complement system consists of a number of small proteins that are synthesized by the liver and is an important contributor to both innate and adaptive immune responses^19^. KEGG PRIMARY BILE ACID BIOSYNTHESIS (G6), REACTOME BILE ACID AND BILE SALT METABOLISM (G9), and REACTOME SYNTHESIS OF BILE ACIDS AND BILE SALTS (G10) are three pathways responsible for the synthesis and metabolism of bile acids and bile salts. The primary bile acids, cholic acid and chenodeoxycholic acid, are synthesized in the liver from cholesterol^20^. Bile salts are ionized bile acids– a more active form. Bile acids and bile salts are critical for digestion and absorption of lipids in the small intestine. KEGG RETINOL METABOLISM (G7) is ranked seventh. Retinol is one of the animal forms of vitamin A and the liver is a particularly rich source of vitamin A^21^.

### Case study 2: breast cancer subtype data

Breast cancer is a heterogeneous disease with different molecular subtypes that are diverse in their natural history and in their responsiveness to treatments^22^. RNA-Seq data from breast cancer patients were downloaded from the data portal of The Cancer Genome Atlas (TCGA). For this data set, we sought to identify pathways linked with estrogen receptor (ER) and progesterone receptor (PGR) activity in breast cancer. These data consist of 69 ER-negative, PGR-negative tumor samples and 162 ER-positive, PGR-positive tumor samples, all from the Stage IIA pathologic group.

The results of RNA-Seq data analyses using the ten gene set-level statistics and with the Signal2Noise gene-level statistic are shown in Supplementary Tables S15–S24. The occurrences and ranks of top 30 pathways over the eight methods are shown in Supplementary Table S25. The top ten pathways with smallest average ranks are listed in Table 2.

Among the top 10 pathways with smallest average ranks, eight pathways, REACTOME DNA STRAND ELONGATION (G1), REACTOME ACTIVATION OF THE PRE REPLICATIVE COMPLEX (G2), REACTOME G1 S SPECIFIC TRANSCRIPTION (G5), REACTOME G1 PHASE (G6), PID ATR PATHWAY (G7), KEGG DNA REPLICATION (G8), REACTOME G2 M CHECKPOINTS (G9), and REACTOME CYCLIN A B1 ASSOCIATED EVENTS DURING G2 M TRANSITION (G10) are related to cell cycle regulation and proliferation. These are well-known pathways altered in cancers. We found that most genes in these pathways are up-regulated in the ER-negative, PGR-negative samples compared to the ER-positive, PGR-positive samples. These results clearly predict that ER-negative, PGR-negative tumors are a more aggressive form of the disease, which is consistent with experimental results that show almost all ER-negative tumors are characterized by increased proliferation^23^. The remaining two pathways, PID FOXM1PATHWAY (G3) and PID AURORA B PATHWAY (G4), are closely related to ER function. The forehead transcription factor (FOXM1) is transcriptionally regulated by ER-alpha and has critical roles in the initiation, progression and drug sensitivity of breast cancer^24^,^25^,^26^,^27^,^28^. Overexpression of aurora kinase A (AURKA) and aurora kinase B (AURKB) has been observed in many types of cancers^29^. Aurora kinases have vital roles in mitosis, and the deregulation of these mitotic kinases may represent an important mechanism driving tumorigenesis^30^,^31^,^32^. Our analyses suggest that the deregulation of FOXM1 and AURKB pathways may contribute to the progression from hormone-dependent to hormone-independent growth of breast cancer since our results show that the activity of both pathways is higher in ER-negative, PGR-negative breast cancer.

**Figure 4:**
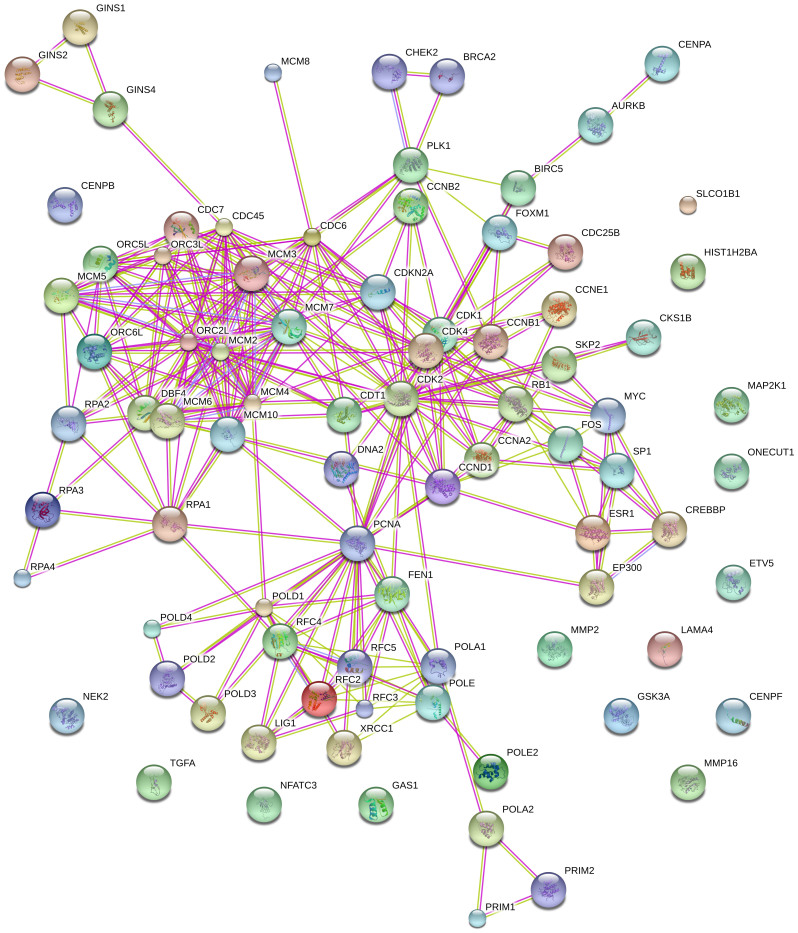
The predicted protein-protein interaction network of protein products of genes in the top three differential pathways associated with breast cancer subtypes (confidence: 0.90). The nodes represent proteins; the edges represent the predicted functional associations. The associations were inferred from two types of evidence from the STRING database: the presence of experimental evidence (purple line) and text-mining evidence (yellow line). Experimental evidence was obtained from protein-protein interaction databases and text-mining evidence from abstracts of scientific literature.

To better understand the relationships between these top pathways, we examined protein-protein interactions (PPIs) between protein products of all genes in the top three pathways based on two types of evidence from the STRING database^33^ (http://string-db.org/): experimental (protein-protein interaction databases) and text-mining (abstracts of scientific literature). The PPI network is shown in Figure 4. Our results indicate that the majority of proteins in the top three pathways are interconnected, which is not unexpected in this case as so many are similarly involved in aspects of the cell cycle. This could explain both how the deregulation of key “hub” genes may affect multiple top pathways, and also how deregulation of distinct genes in multiple samples may have the same phenotypic effect if they act on a similar set of genes in key pathways.

In summary, our results show: (1) analyses of diverse tissue samples not only identified well-known trait-associated pathways but also provided potentially novel insights into the molecular mechanisms of complex traits and human disease; (2) results were highly reproducible over multiple analytical methods for the two data sets we analyzed.

### Comparison to existing tools

Recently, several tools have been developed for differential expression analysis of individual genes for RNA-Seq data, for example DESeq^34^, edgeR^35^, NOISeq^36^, and Cuffdiff^37^. These tools generate a list of scores or p-values indicating the correlation of each gene with a phenotype difference. Any suitable gene set-level metric can then be used to study the associations between gene sets and phenotype based on this list. However, the sample permutation strategy is not applicable to methods that take as input a list of scores or p-values generated using other tools. Therefore, list-based approaches usually assess statistical significance of association signals by gene permutation - shuffling gene labels. It is common for genes in a pathway to have correlated expression profiles. Sample permutation preserves these correlation structures within gene sets, and so likely provides a more accurate background model than gene permutation. We believe this enables GSAASeqSP to generate more accurate null distributions for gene-level and gene set-level statistics in expression-based gene set analysis since it uses sample permutation to preserve correlation information during randomization.

To our knowledge, the pipeline for DE-only analysis in SeqGSEA^12^ is so far the only published tool for sample permutation-based gene set association analysis for RNA-Seq gene expression data. SeqGSEA uses the Weighted_KS statistic as gene set-level statistic, which we evaluated in our simulation study as it is also implemented in GSAASeqSP. The DE-only analysis in SeqGSEA uses DESeq^34^ for gene-level differential expression analysis. DESeq and edgeR^35^ are two popular Bioconductor packages that test for gene-level differential expression in RNA-Seq based on the negative binomial (NB) distribution. We have explored using these tools in another toolset for gene set association analysis of RNA-Seq data, Gene Set Association Analysis for RNA-Seq with Gene Permutation (GSAASeqGP). GSAASeqGP contains the gene-level differential expression metrics proposed by edgeR and DESeq and uses the Weighted_KS statistic as gene set-level statistic (Supplementary Table S1). Currently, GSAASeqGP uses the gene permutation strategy. However, we also implemented this with sample permutation (called “GSAASeqSPNB”). We found that the run time for GSAASeqSPNB is unacceptable, as we describe in more detail below. In addition, we have implemented the gene permutation strategy for each analytical method in GSEASeqSP (called “GSAASeqSPGP”).

We carried out comparisons between the two NB-based metrics, DESeq and edgeR, in GSAASeqGP and the Signal2Noise metric in GSAASeqSP using the gene permutation strategy. Here, we chose the S5 simulation scenario to evaluate these since it effectively measures the ability of methods to detect association signals. We chose Signal2Noise as the gene-level statistic due to its superior performance in the simulation studies. The results of these tests are shown in Supplementary Tables S26–S27, which include the average run time, RR, p-value, FDR, FWER, and power over 200 replicates. For the comparison of run times, we also included a predicted run time for DESeq/edgeR-based GSAASeqSPNB, which was based on the time of running a single gene-level analysis. Let *T*(*GSAASeqGP*) be the total time for running DESeq-based GSAASeqGP with *N* permutations and *T*(*DESeq*) be the time for running a single DESeq analysis, then the total run time for running DESeq-based GSAASeqSPNB, *T*(*GSAASeqSPNB*), can be calculated as *T*(*GSAASeqSPNB*) = (*T*(*DESeq*) * *N*) + (*T*(*GSAASeqGP*) − *T*(*DESeq*)). For gene permutations, we just need to run DESeq one time while for sample permutation, we have to run DESeq *N* times. We set *N* to 2000 in our simulation study. In addition, we also included the results from GSAASeqSP with Signal2Noise as gene-level statistic - see Supplementary Tables S26–S27 for details.

Based on a comparison of run times (Supplementary Table S26), our results show: (1) using the gene permutation strategy, methods in GSAASeqSPGP are faster than methods in GSAASeqGP; and (2) using the sample permutation strategy, methods in GSAASeqSP are much faster than methods in GSAASeqSPNB. The DE-only analysis in SeqGSEA is very similar to DESeq-based GSAASeqSPNB (called GSAASeqSPNB_DESeq:Weighted_KS), as both use DESeq for differential gene expression analysis, Weighted_KS for gene set analysis, and a sample permutation strategy. We predict that the run times of SeqGSEA and GSAASeqSPNB_DESeq:Weighted_KS will be similar. Based on our calculations (Supplementary Table S26), GSAASeqSPNB_DESeq:Weighted_KS (3531661 secs) is approximately 75142 times slower than GSAASeqSP with Signal2Noise and Weighted_KS as gene-level and gene set-level statistics (called GSAASeqSP_Signal2Noise:Weighted_KS) (47 secs) when using 2000 permutations to generate null distributions. Namely, GSAASeqSP_Signal2Noise:Weighted_KS takes approximately 16 hours to finish running on all of our simulated datasets while GSAASeqSPNB_DESeq:Weighted_KS would need approximately 134 years. These analyses imply that DESeq may be more suited for gene permutation-based gene set analysis.

Overall, our performance comparisons (Supplementary Table S27) indicate: (1) when using Weighted_KS as the gene set-level statistic and employing the gene permutation strategy, Signal2Noise performed slightly better than DESeq with respect to RRs while DESeq is slightly better than Signal2Noise with respect to FDRs. The RRs and FDRs for GSAASeqSPGP_Signal2Noise:Weighted_KS, GSAASeqGP_DESeq:Weighted_KS, and GSAASeqGP_edgeR:Weighted_KS are 0.98, 0.96, 0.93 and 0.030236, 0.027124, 0.066553, respectively; (2) sample permutation performed better than or the same as gene permutation for all combinations of Signal2Noise gene-level statistic and eight gene set-level statistics in GSAASeqSP with respect to RRs and FDRs.

## Discussion

In this study, we describe GSAASeqSP, a novel toolset that we developed for gene set association analysis of sequence count data. This toolset contains a comprehensive set of analytical methods through combinations of multiple gene-level statistics and multiple gene set-level statistics. We rigorously evaluated the ability of these methods to identify association signals using both simulated and real data. In this paper, our results focused on pathways robustly identified as top pathways by at least four methods. Most pathways identified through this strategy have well-established roles in the relevant complex trait. In addition, results from each method alone may also generate meaningful biological insights. For instance, the PID PLK1 PATHWAY was ranked fourth by the combined Signal2Noise (gene-level):Weighted_KS (gene set-level) method. In this pathway, many genes, such as polo-like kinase 1 (PLK1), are up-regulated in ER-negative, PGR-negative breast cancer. PLK1 is a potential therapeutic target for the treatment of the poor prognosis-associated triple-negative breast cancer (TNBC) since it was found to be significantly overexpressed in TNBC compared with the other breast cancer subtypes^38^,^39^.

GSAASeqSP currently includes three statistics for gene differential expression analysis and eight statistics for gene set analysis. Among these statistics, some have not been previously used in gene set analyses, while the majority has been used in conjunction with microarray data. However, except for Weighted_KS adopted by SeqGSEA^11^,^12^, the performance of these statistics on RNA-Seq data had not been evaluated. Microarray data is approximately normally distributed while RNA-Seq data follows a NB distribution, so a statistic that works well for microarray data analysis may fail to identify signals in RNA-Seq data - the MinP statistic is an example. Using simulations, we have comprehensively evaluated the performance of different analytical methods under various scenarios. Our results show that most methods captured signals embedded in the simulated count data effectively. Since each method has its own advantages and disadvantages, we suggest that users evaluate multiple methods when analyzing their data. We provide many options for solving the same problem in order that users can compare and determine which one(s) are best for their specific purposes. In addition, in the simulation study we presented results for all combinations of gene-level statistics and gene set-level statistics, but we are aware that a few of combinations may not be statistically sound. However, these types of combinations generally performed poorly so they can be ignored in practice. Our simulation results provide guidance on the selection of appropriate combinations.

The advantages of GSAASeqSP from the point of view of computation include: 1) it is computationally efficient; GSAASeqSP took approximately 0.3, 0.8, 2.8, 2.5, 2.7, 1.1, 1.0 and 1.5 hrs for Weighted_KS, L2Norm, Mean, WeigtedSigRatio, SigRatio, GeometricMean, FisherMethod, RankSum, respectively, in the analysis of breast cancer data using one computational node (Intel(R) Xeon(R) CPU X5650 @ 2.67 GHz) on a Linux cluster; 2) GSAASeqSP can be run from both the command line and the graphical user interface (GUI) making it is user-friendly; and 3) GSAASeqSP is implemented using a flexible modular structure allowing it to be easily extended to include new statistics in the future.

## Methods

GSAASeqSP takes as input raw count data from multiple samples, *a priori* defined gene sets, and phenotype labels of samples. Its workflow includes 1) normalization of raw count data; 2) differential expression analysis of individual genes; 3) gene set association analysis; 4) assessment of statistical significance of associations (Figure 1). The details of each step are described below:

### Normalization of raw count data

Normalization is very important for gene expression analysis as studies have shown that gene set analysis can be affected by both systematic biases and technical biases inherent to RNA-Seq technology, such as between-sample differences (i.e. library size)^40^ and within-sample gene-specific effects (i.e. gene length)^41^. Normalization enables accurate comparisons of expression levels between and within samples by adjusting for these biases. There are several methods available for normalizing RNA-Seq data. In GSAASeqSP, we normalize raw counts using the same method implemented in the DESeq Bioconductor package^34^. Dillies et al.^40^ comprehensively evaluated a series of normalization methods and their results show that the DESeq normalization and Trimmed Mean of M values (TMM) implemented in the edgeR Bioconductor package^35^ outperformed the other methods compared. To avoid zero counts, we added 1 to all counts in the data set before normalization.

### Differential expression analysis

Three statistics were evaluated for differential expression analysis of individual genes: Signal2Noise, log2Ratio, and Signal2Noise_log2Ratio. Signal2Noise is the primary gene-level statistic used by GSEA^2^, one of the most popular tools for gene set enrichment analysis of microarray data. Log2Ratio is a commonly used metric for differential expression analysis of microarray data as well. In addition, we developed a new statistic, Signal2Noise_log2Ratio, by modifying an existing statistic introduced by NOISeq^36^, software designed to perform differential expression analysis of individual genes for RNA-Seq data. A detailed description of these statistics is available in the Supplementary Material. GSAASeqSP employs a sample-based permutation procedure to assess the statistical significance of associations, and this is achieved by shuffling the phenotype labels of samples and recalculating the test statistics many times. Compared with methods that instead permute the genes, sample permutation-based approaches generate more accurate null distributions for gene-level and gene set-level statistics in expression-based gene set analysis since the expression profiles of genes in biological pathways are usually correlated. The sample permutation preserves the gene-gene correlation structures during the randomization, thus, phenotypic associations can be examined more accurately. In this step, a differential expression score and a p-value are computed for each gene for both the observed data and permutations.

### Gene set association analysis

#### Computation of gene set association scores

Ten statistics were evaluated for gene set association analysis: Weighted_KS, L2Norm, Mean, WeightedSigRatio, SigRatio, GeometricMean, TruncatedProduct, FisherMethod, MinP, and RankSum. A detailed description of these statistics is available in the Supplementary Material. These statistics can be divided into three categories: score based (Weighted_KS, L2Norm, Mean, WeightedSigRatio, SigRatio), p-value based (GeometricMean, TruncatedProduct, FisherMethod, MinP), and rank based (RankSum). Among these statistics, Weighted_KS, L2Norm, Mean, GeometricMean, TruncatedProduct, FisherMethod, MinP, and RankSum have already been used for gene set analysis of microarray data. Here we adapted these statistics for and evaluated their performance for the first time on RNA-Seq count-based data. WeightedSigRatio and SigRatio are novel and have not been previously applied to gene set analysis. In this step, a gene set association score (AS) is computed for each gene set for both the observed data and permutations based on any of the ten gene set-level statistics. The differential expression scores or p-values of individual genes can be computed by any of the three gene-level statistics: Signal2Noise, log2Ratio, or Signal2Noise_log2Ratio.

#### Normalization of gene set association scores

To correct for possible heterogeneity of information at each gene set, for example differences in the number of genes in the gene set or correlation structure, we normalize the AS by the mean of its null distribution generated by permutations. For a particular gene set *S*, given its actual AS *AS*_0 _and ASs calculated from permutations *π* = 1,…,*N* {*AS*_1_,…,*AS_N_*}, the normalized association score (NAS) is computed as 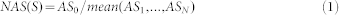
This normalization method was originally introduced by GSEA^2^.

### Assessment of statistical significance and adjustment for multiple hypothesis testing

Statistical significance refers to the probability that a difference observed between groups occurs by chance. We assess the statistical significance of the AS and adjust for multiple hypothesis testing based on a sample-based permutation procedure. The null distribution of the AS for a particular gene set is generated by shuffling the phenotypic class labels and recalculating the AS many times. This procedure effectively preserves the correlation structure in the gene set. Consider a particular gene set *S*, suppose *AS*_0_ is the actual AS and {*AS*_1_,…,*AS_N_*} are the ASs for permutations *π* = 1,…,*N*, the p-value for the gene set *S* from the Weighted_KS, L2Norm, Mean, WeightedSigRatio, SigRatio, or FisherMethod test is computed as 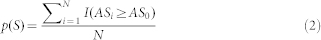
while the p-value for GeometricMean, TruncatedProduct, MinP, or RankSum is computed as 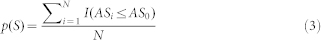
Where the indicator variables *I*(*AS_i_* ≥ *AS*_0_) and *I*(*AS_i_* ≤ *AS*_0_) equal 1 if *AS_i_* ≥ *AS*_0 _and *AS_i_* ≤ *AS*_0 _respectively otherwise they are 0. Smaller p-values indicate higher probability that a gene set is associated with the phenotype.

We use the false discovery rate (FDR) and the family-wise error rate (FWER) based on NAS to correct for multiple hypothesis testing and to control the proportion of false positives below a certain threshold. Given *m* gene sets {*S*_1_,…,*S_m_*} and label permutations *π* = 1,…,*N*, the FDR for the gene set *S_i_*from the Weighted_KS, L2Norm, Mean, WeightedSigRatio, SigRatio, or FisherMethod test is computed as 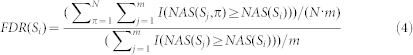
The FDR for GeometricMean, TruncatedProduct, MinP, or RankSum is computed as 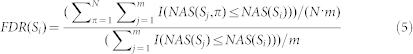
Where *NAS*(*S_j_*,*π*) is the *NAS* for gene set *j* with label permutation *π*. *NAS*(*S_j_*) is the *NAS* for gene set *j*. The indicator variables *I*(*NAS*(*S_j_*,*π*) ≥ *NAS*(*S_i_*)), *I*(*NAS*(*S_j_*) ≥ *NAS*(*S_i_*)), *I*(*NAS*(*S_j_*,*π*) ≤ *NAS*(*S_i_*)), and *I*(*NAS*(*S_j_*) ≤ *NAS*(*S_i_*)) equal 1 if *NAS*(*S_j_*,*π*) ≥ *NAS*(*S_i_*), *NAS*(*S_j_*) ≥ *NAS*(*S_i_*), *NAS*(*S_j_*,*π*) ≤ *NAS*(*S_i_*), and *NAS*(*S_j_*) ≤ *NAS*(*S_i_*) respectively otherwise they are 0.

The FWER for the gene set *S_i_*from the Weighted_KS, L2Norm, Mean, WeightedSigRatio, SigRatio, or FisherMethod test is computed as 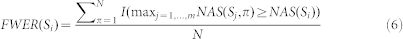
The FWER for GeometricMean, TruncatedProduct, MinP, or RankSum is computed as 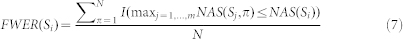
Where the indicator variables *I*(max*_j_*_ = 1,…,*m*_*NAS*(*S_j_*,*π*) ≥ *NAS*(*S_i_*)) and *I*(max*_j_*_ = 1,…,*m*_*NAS*(*S_j_*,*π*) ≤ *NAS*(*S_i_*)) are 1 if max*_j_*_ = 1,…,*m*_*NAS*(*S_j_*,*π*) ≥ *NAS*(*S_i_*) and max*_j_*_ = 1,…,*m*_*NAS*(*S_j_*,*π*) ≤ *NAS*(*S_i_*) respectively otherwise they are 0.

### Generation of simulated data

To evaluate the effectiveness of different gene-level and gene set-level statistics, we conducted a comprehensive simulation study. We designed 6 scenarios of differential expression. For each scenario, 200 data sets were independently generated from the same statistical model. In each data set, we simulated 200 samples corresponding to one phenotype and 200 samples corresponding to a second phenotype. For each sample, we simulated RNA-seq read counts for 1000 genes. In our simulations, we assume that the expression differences observed between the two phenotypes result from genotypic differences. Based on this assumption, we first simulated the genetic association between gene sets and phenotype then simulated the differential expression corresponding to the genetic association. Simulating gene expression variation based on genetic variation makes simulated data closer to the real data than simulating gene expression variation independently, since genetic variants are one of the major causes of differential gene expression^42^,^43^. For further details on generating these data, please see the Supplementary Material.

## Author Contributions

Q.X., S.M. and T.S.F. conceived the study; Q.X. designed the method, wrote the software, performed experiments; Q.X., S.M. and T.S.F. analyzed results and wrote the manuscript.

## Supplementary Material

Supplementary Information Supplementary Material

Supplementary Information Supplementary Tables S2-S27

## Figures and Tables

**Table 1:** The occurrences and ranks of the top pathways across eight methods associated with differences between kidney and liver tissue

Index	Pathway	NOC	Rank 1	Rank 2	Rank 3	Rank 4	Rank 5	Rank 6	Rank 7	Rank 8	Avg
G1	BIOCARTA AMI PATHWAY	8	1	2	7	1	15	1	1	2	3.75
G2	REACTOME XENOBIOTICS	8	3	1	6	3	23	2	2	1	5.13
G3	REACTOME COMPLEMENT CASCADE	6	0	4	9	0	6	4	9	4	6.00
G4	BIOCARTA INTRINSIC PATHWAY	8	2	3	8	2	26	3	3	3	6.25
G5	BIOCARTA COMP PATHWAY	5	0	10	1	0	0	8	6	8	6.60
G6	KEGG PRIMARY BILE ACID BIOSYNTHESIS	6	4	6	12	0	0	10	8	7	7.83
G7	KEGG RETINOL METABOLISM	6	11	8	13	0	0	6	4	6	8.00
G8	KEGG COMPLEMENT AND COAGULATION CASCADES	6	12	11	10	0	0	9	7	5	9.00
G9	REACTOME BILE ACID AND BILE SALT METABOLISM	6	0	9	11	0	10	5	10	9	9.00
G10	REACTOME SYNTHESIS OF BILE ACIDS AND BILE SALTS	7	9	5	14	0	18	7	5	15	10.43

NOC: number of occurrences; 1: Weighted_KS; 2: L2Norm; 3: Mean; 4: WeigtedSigRatio; 5: SigRatio; 6: GeometricMean; 7: FisherMethod; 8: RankSum; Avg: the average rank.

**Table 2:** The occurrences and ranks of top pathways across eight methods associated with differences in breast cancer subtypes

Index	Pathway	NOC	Rank 1	Rank 2	Rank 3	Rank 4	Rank 5	Rank 6	Rank 7	Rank 8	Avg
G1	REACTOME DNA STRAND ELONGATION	7	0	5	4	2	4	1	1	2	2.71
G2	REACTOME ACTIVATION OF THE PRE REPLICATIVE COMPLEX	8	2	9	2	4	6	3	3	19	6.00
G3	PID FOXM1PATHWAY	8	1	2	3	10	20	5	4	5	6.25
G4	PID AURORA B PATHWAY	8	3	4	11	1	13	4	5	25	8.25
G5	REACTOME G1 S SPECIFIC TRANSCRIPTION	8	9	7	1	18	12	2	2	16	8.38
G6	REACTOME G1 PHASE	8	11	6	8	15	21	8	7	4	10.00
G7	PID ATR PATHWAY	7	0	19	14	3	5	9	11	13	10.57
G8	KEGG DNA REPLICATION	8	8	16	10	5	8	11	10	18	10.75
G9	REACTOME G2 M CHECKPOINTS	8	18	8	5	25	1	13	9	7	10.75
G10	REACTOME CYCLIN A B1 ASSOCIATED EVENTS DURING G2 M TRANSITION	7	10	1	12	16	0	6	6	29	11.43
