# Supplementary material for: GSAASeqSP: A Toolset for Gene Set Association Analysis of RNA-Seq Data
Source: Sci Rep. 2014 Sep 12;4:6347. doi: 10.1038/srep06347 (PMC4161965; doi:10.1038/srep06347)
Supplement: Supplementary Information — Supplementary Material [file srep06347-s1.pdf]

Supplementary Material

GSAASeqSP: A Toolset for Gene Set Association Analysis of RNA-Seq Data

Qing Xiong[1,*], Sayan Mukherjee[2] & Terrence S. Furey[3,*]

[1]Department of Computer Science and Technology, Department of Statistics, Southwest University, Chongqing 400715, China;

[2]Department of Statistical Science, Department of Computer Science, and Department of Mathematics, Duke University, Durham, NC 27708, USA;

[3]Department of Genetics, Department of Biology, Lineberger Comprehensive Cancer Center, and Carolina Center for Genomics and Society, The University of North Carolina at Chapel Hill, Chapel Hill, NC 27599, USA.

1 Tables

Table S1. A list of computational tools for gene set analysis of two sample groups

Tool	Description	Reference
GSAASeqSP	**Gene set association analysis for RNA-Seq data based on sample permutation.** GSAASeqSP includes three statistics (Signal2Noise, log2Ratio, Signal2Noise_log2Ratio) for gene differential expression analysis and eight statistics (Weighted_KS, L2Norm, Mean, WeightedSigRatio, SigRatio, GeometricMean, FisherMethod, RankSum) for gene set association analysis. GSAASeqSP is a pure Java application, and it runs fast.	http://gsaa.unc.edu/login/userguide_gsaaseqsp.html
GSAASeqGP	**Gene set association analysis for RNA-Seq data based on gene permutation.** GSAASeqGP uses several existing R packages (edgeR[1], DESeq[2], NOISeq[3], DEGseq[4], baySeq[5]) for gene differential expression analysis, and transform the output (p-values or probabilities) from these packages into ranks, and then takes as input these ranks for gene set association analysis, which is based on a weighted Kolmogorov-Smirnov test.	http://gsaa.unc.edu/login/userguide_gsaaseqgp.html
GSAA	**Integrative gene set association analysis of microarray gene expression data and SNP data.** GSAA includes five statistics (ChiSquare_Geno, ChiSquare_Allele, Diff_of_Alleles, tTest_Geno, tTest_Allele) for single-SNP association analysis, one statistic (Maximum) for SNP set association analysis, three statistics (Signal2Noise, tTest, log2Ratio) for gene differential expression analysis, three statistics (ZScore_Sum, Fisher_Method, Rank_Sum) for integrative gene association analysis, and	Genome Res. (Xiong, et al., 2012)[6]

	one statistic (Weighted_KS) for gene set association analysis.	
GSAA-SNP	**Gene set association analysis for SNP data.** GSAA-SNP includes five statistics (ChiSquare_Geno, ChiSquare_Allele, Diff_of_Alleles, tTest_Geno, tTest_Allele) for single-SNP association analysis and one statistic (Weighted_KS) for gene set association analysis.	http://gsaa.unc.edu/login/userguide_gsaasnp.html
GSEA	**Gene set enrichment analysis for microarray gene expression data.** GSEA includes eight statistics (Signal2Noise, tTest, Cosine, Euclidean, Manhatten, Pearson, Ratio_of_Classes, Diff_of_Classes) for gene differential expression analysis and one statistic (Weighted_KS) for gene set enrichment analysis.	Proc Natl Acad Sci U S A. (Subramanian, et al., 2005)[7]
SeqGSEA	**Integrative gene set enrichment analysis of differential expression and splicing for RNA-Seq data.** SeqGSEA designed an additional tool for gene set enrichment analysis purely based on differential expression – the DE-only analysis. This tool uses an existing R package, DESeq[2], for gene differential expression analysis, and uses the Weighted_KS statistic for gene set enrichment analysis.	BMC Bioinformatics. (Wang, et al., 2013)[8] Bioinformatics (Wang, et al., 2014)[9]

2 Methods

2.1 Differential expression analysis

In GSAASeqSP, we provide three statistics for differential expression analysis of individual genes: Signal2Noise, log2Ratio, and Signal2Noise_log2Ratio. GSAASeqSP employs a sample-based permutation procedure to assess the association significance, so in this step a differential expression score and a p-value are computed for each gene for both the observed data and permutations.

Consider two phenotype classes, C_1 and C_2:

(1) Signal2Noise is the absolute value of the difference of the class means scaled by the standard deviation

$$s_i = \left| \frac{\mu_i^1 - \mu_i^2}{\sigma_i^1 + \sigma_i^1} \right| \qquad (1)$$

Where (μ_i^1, μ_i^2) and (σ_i^1, σ_i^2) are the means and standard deviations of expression values of gene i in classes C_1 and C_2, respectively. Each s-score is normalized by the sum of s-scores of all genes in the data set. The value of the statistic represents the extent to which a gene is differentially expressed between two phenotypic classes; bigger value indicates higher differential expression.

To estimate p-values, we first generate the gene-wise null distribution of the Signal2Noise statistic by a sample-based permutation procedure, and we then calculate a p-value for the test statistic based on its null distribution. Suppose s_0 is the value of the Signal2Noise statistic for the observed data and $\{s_1,...,s_N\}$ are the values for permutations $\pi = 1,...,N$. The p-value for the Signal2Noise statistic is computed as

$$p(s_i) = \frac{\sum_{j=0}^{N} I(s_j \geq s_i)}{N+1} \qquad (2)$$

Where $I(s_j \geq s_i)$ is an indicator variable that is one if $s_j \geq s_i$ and is otherwise zero. Smaller p-value indicates higher probability that a gene is differentially expressed between two phenotypic classes.

(2) log2Ratio is the absolute value of the log2 ratio of the class means

$$r_i = |\log_2(\frac{\mu_i^1}{\mu_i^2})| \qquad (3)$$

Where (μ_i^1, μ_i^2) are the means of expression values of gene i in classes C_1 and C_2, respectively. Each r-score is normalized by the sum of r-scores of all genes in the data set. The value of the statistic represents the extent to which a gene is differentially expressed between two phenotypic classes; bigger value indicates higher differential expression.

We first generate the gene-wise null distribution of the log2Ratio statistic by a sample-based permutation procedure, and we then estimate a p-value for the test statistic based on its null distribution. Suppose r_0 is the value of the log2Ratio statistic for the observed data and $\{r_1,...,r_N\}$ are the values for permutations $\pi = 1,...,N$. The p-value for the log2Ratio statistic is computed as

$$p(r_i) = \frac{\sum_{j=0}^{N} I(r_j \geq r_i)}{N+1} \qquad (4)$$

Where $I(r_j \geq r_i)$ is an indicator variable that is one if $r_j \geq r_i$ and is otherwise zero. Smaller p-value indicates higher probability that a gene is differentially expressed between two phenotypic classes.

(3) Signal2Noise_log2Ratio is the mean of Signal2Noise and log2Ratio

We first generate the gene-wise joint null distribution of the Signal2Noise statistic and log2Ratio statistic by a sample-based permutation procedure, and we then calculate a gene's differential expression score and its p-value based on this null distribution. Suppose s_0 and r_0 are the values of the Signal2Noise statistic and log2Ratio statistic for the observed data, respectively. $\{s_1,...,s_N\}$ and $\{r_1,...,r_N\}$ are the values for permutations $\pi = 1,...,N$. These s-scores and r-scores are from different types of tests and thus have different scales. In order to bring these scores to a common scale, we convert them to standard scores. The standard s-scores $\{z_0^s,...,z_N^s\}$ are computed as

$$z_i^s = \frac{s_i - \mu_s}{\sigma_s} \qquad (5)$$

and the standard r-scores $\{z_0^r,...,z_N^r\}$ are similarly computed as

$$z_i^r = \frac{r_i - \mu_r}{\sigma_r} \qquad (6)$$

Where (μ_s, μ_r) and (σ_s, σ_r) are the means and standard deviations of the null distributions corresponding to s_i and r_i, respectively. The Signal2Noise_log2Ratio statistic is then defined by

$$sr_i = \frac{(z_i^s + c) + (z_i^r + c)}{2} \qquad (7)$$

Where c is the minimum score of all standard s-scores and standard r-scores of all genes over the observed data and all permutations. c is used to convert all z-scores to positive values. The value of the statistic represents the extent to which a gene is differentially expressed between two phenotypic classes; bigger value indicates higher differential expression. The p-value from the joint distribution is computed as

$$p(z_i^s, z_i^r) = \frac{\sum_{j=0}^{N} I(z_j^s \geq z_i^s, z_j^r \geq z_i^r)}{N+1} \qquad (8)$$

Where $I(z_j^s \geq z_i^s, z_j^r \geq z_i^r)$ is an indicator variable that is one if $z_j^s \geq z_i^s$ and $z_j^r \geq z_i^r$, and is otherwise zero. This statistic was created by modifying an existing statistic introduced by NOISeq[3]. Smaller p-value indicates higher probability that a gene is differentially expressed between two phenotypic classes.

2.2 Gene set association analysis

2.2.1 Computation of gene set association scores

Ten statistics are evaluated for gene set association analysis of RNA-Seq data: Weighted_KS, L2Norm, Mean, WeightedSigRatio, SigRatio, GeometricMean, TruncatedProduct, FisherMethod, MinP, and RankSum. These statistics can be divided into three categories: score based (Weighted_KS, L2Norm, Mean, WeightedSigRatio, SigRatio), p-value based (GeometricMean, TruncatedProduct, FisherMethod, MinP) and rank based (RankSum). In this step, for a particular gene set S including H genes, given the differential expression scores $\{g_1,...,g_H\}$ and the corresponding p-values $\{p_1,...,p_H\}$ for all genes in the gene set, a gene set association score (AS) is computed for both the observed data and permutations based on any of the following gene set-level statistics. The differential expression scores and p-values can be computed by any of the above three gene-level statistics: Signal2Noise, log2Ratio, or Signal2Noise_log2Ratio.

(1) Weighted Kolmogorov-Smirnov test (Weighted_KS)

Given the differential expression scores of all genes in the data set, a weighted Kolmogorov-Smirnov (KS) test is used to determine the extent to which a gene set is associated with a given phenotype. This test was originally proposed by Subramanian et al[7]. Essentially, the weighted KS test determines for each gene set whether the genes belonging to that gene set are preferentially near the top of the ranked ordered list based on differential expression scores. More specifically, for gene set S, given the differential expression scores $\{g_1,...,g_N\}$ for all genes in the data set, a running association score $RAS_S(i)$ for the rank ordered genes in positions $i=1,...,N$ is computed as

$$RAS_S(i) = \frac{1}{N_S}\sum_{j=1}^{i} g_j I(j\in S) - \frac{1}{N-H}\sum_{j=1}^{i} I(j\notin S),\ N_S = \sum_{j=1}^{N} g_j I(j\in S), \quad (9)$$

Where $I(j\in S)$ is an indicator variable that is one if the jth gene in the rank ordered list is in gene set S and is otherwise zero. Similarly, $I(j\notin S)$ takes the value of zero if the jth gene is in the gene set and is otherwise one. The association score of gene set S, $AS(S)$, is the maximum deviation from zero of the running association score over the positions $i=1,...,N$

$$AS(S)_+ = \max_{i=1,...,N}[RAS_S(i)],\ AS(S)_- = \min_{i=1,...,N}[RAS_S(i)]. \quad (10)$$

Finally, if $|AS(S)_+| > |AS(S)_-|$ then the final gene set association score $AS(S) = AS(S)_+$, otherwise $AS(S) = AS(S)_-$.

The AS indicates the strength of the association between the gene set and the phenotype, bigger value indicates stronger association. The differential expression scores we used lack directionality, so a negative AS(S) means there is no association between the gene set and the phenotype. We set AS(S) = 0.0001 if AS(S)<0 so that negative AS will not confuse the interpretation.

(2) L_2-norm (L2Norm)

We here use the L_2-norm of the differential expression scores of all genes in a gene set as the test statistic to evaluate the association of that gene set with the phenotype. This statistic has been adopted as the gene set-level statistic for analyzing microarray data by SAM-GS[10], The association score of the gene set S based on the L_2-norm of differential expressions is computed as

$$AS(S) = \sum_{i=1}^{H} g_i^2 \qquad (11)$$

The AS indicates the strength of the association between the gene set and the phenotype, bigger value indicates stronger association.

(3) Mean (Mean)

The simple mean statistic tests whether the mean of differential expression scores in the gene set from the observed data is bigger than what would be expected from random samples. The association score of the gene set S based on the mean test is computed as

$$AS(S) = \frac{\sum_{i=1}^{H} g_i}{H} \qquad (12)$$

The AS indicates the strength of the association between the gene set and the phenotype, bigger value indicates stronger association.

(4) Weighted Significance Ratio (WeightedSigRatio)

The Weighted Significance Ratio (WSR) is defined as the ratio of the sum of significant differential expression scores to the sum of all differential expression scores in the gene set. The association score of the gene set S based on the WSR test is computed as

$$AS(S) = \frac{\sum_{i=1}^{H} g_i I(p_i \le \tau)}{\sum_{i=1}^{H} g_i} \qquad (13)$$

Where τ is the p-value threshold that is used as a cutoff for determining significance. τ is a parameter that can be specified by users in GSAASeqSP. In this study, we set $\tau = 0.05$; $I(p_i \le \tau)$ is an indicator variable that is one if $p_i \le \tau$, and is otherwise zero. The AS indicates the strength of the association between the gene set and the phenotype, bigger value indicates stronger association.

(5) Significance Ratio (SigRatio)

The Significance Ratio (SR) test compares the proportion of significant genes to all genes in the gene set. The association score of the gene set S based on the SR test is computed as

$$AS(S) = \frac{\sum_{i=1}^{H} I(p_i \le \tau)}{H} \qquad (14)$$

Where τ is the p-value cutoff, it was set to 0.05 in this study. τ is a parameter that can be specified by users in GSAASeqSP. $I(p_i \le \tau)$ is an indicator variable that is one if $p_i \le \tau$, and is otherwise zero. The AS indicates the strength of the association between the gene set and the phenotype, bigger value indicates stronger association.

(6) Geometric mean (GeometricMean)

The geometric mean (GM) is defined as the nth root of the product of n numbers. Here we use the geometric mean of the p-values over all genes in a gene set as the test statistic to evaluate the association of that gene set with the phenotype. The association score of the gene set S based on the GM test is computed as

$$AS(S) = \sqrt[H]{p_1 p_2 ... p_H} \qquad (15)$$

The AS indicates the strength of the association between the gene set and the phenotype, smaller value indicates stronger association.

(7) Truncated product method (TruncatedProduct)

Truncated product method (TPM)[11] is a global test that combines p-values from several hypothesis tests to provide a p-value for the overall hypothesis that all single hypotheses are true. It has several advantages over the ordinary Fisher product test. Here we use TPM test statistic to combine p-values of genes in a gene set into an overall significance level to investigate the association of that gene set with the phenotype. The association score of the gene set S based on the TPM is computed as

$$AS(S) = \prod_{i=1}^{H} p_i I(p_i \le \tau) \qquad (16)$$

Where τ is the p-value cutoff, it was set to 0.05 in this study. $I(p_i \le \tau)$ is an indicator variable that is one if $p_i \le \tau$, and is otherwise zero. The AS indicates the strength of the association between the gene set and the phenotype, smaller value indicates stronger association.

(8) Fisher's method (FisherMethod)

Fisher's method is widely used to combine p-values from multiple independent tests. Here we use Fisher's method to summarize p-value evidence from all genes in a gene set into an overall significance level. The association score of the gene set S based on the Fisher product test is computed as

$$AS(S) = -2\sum_{i=1}^{H} \log_e p_i \qquad (17)$$

The AS indicates the strength of the association between the gene set and the phenotype, smaller value indicates stronger association.

(9) Minimum p-value (MinP)

We modified a MinP statistic[12] and use it as the test statistic for gene set association analysis. For gene set S, given the p-value matrix $\{p_{10},...,p_{1N};...;p_{H0},...,p_{HN}\}$ $p_{10} \le ... \le p_{H0}$ of all genes in S calculated from the observed data and permutations $\pi = 1,...,N$, the association score based on the MinP method is computed as

$$AS(S_i) = \min_{j=1,\ldots,H}[\sum_{m=0}^{N} I(\prod_{k=1}^{j} p_{km} \leq \prod_{k=1}^{j} p_{ki}) \Big/ (N+1)] \quad \text{for} \quad i = 0,\ldots,N \qquad (18)$$

Where $I(\prod_{k=1}^{j} p_{km} \leq \prod_{k=1}^{j} p_{ki})$ is an indicator variable that is one if $\prod_{k=1}^{j} p_{km} \leq \prod_{k=1}^{j} p_{ki}$, and is otherwise zero. The AS indicates the strength of the association between the gene set and the phenotype, smaller value indicates stronger association.

(10) Rank Sum (RankSum)

The Rank Sum is the sum of ranks of all gens in the gene set. We first transform the differential expression scores $\{g_1,\ldots,g_N\}$ of all genes in the data set into ranks $\{r_1,\ldots,r_N\}$, the association score of the gene set S based on the Rank Sum statistic is therefore defined as

$$AS(S) = \sum_{i=1}^{H} r_i \qquad (19)$$

The AS indicates the strength of the association between the gene set and the phenotype, smaller value indicates stronger association.

2.2.2 Normalization of gene set association scores

To correct for possible heterogeneity of information at each gene set, for example differences in the gene set size or correlation structure, we normalize the AS by the mean of its null distribution generated by permutations. For a particular gene set S, given its actual AS AS_0 and ASs calculated from permutations $\pi = 1,\ldots,N$ $\{AS_1,\ldots,AS_N\}$, the normalized association score (NAS) is computed as

$$NAS(S) = AS_0 \big/ mean(AS_1,\ldots,AS_N) \qquad (20)$$

This normalization method was originally introduced by GSEA[7]. The normalized association scores are then used for downstream analyses.

2.3 Generation of simulated data

To evaluate the effectiveness of different gene-level and gene set-level statistics, we conducted a comprehensive simulation study. We designed 6 scenarios of differential expression. For each scenario, 200

case-control data sets were independently generated from the same statistical model. In each data set, we simulated 200 cases and 200 controls, and for each case and control, we simulated the read counts of 1000 genes. It is well known that genetic variants are one of the major determinants that cause differential expression[13,14]. Therefore, in our simulations, we assume that the expression difference observed between cases and controls are the results from genotypic difference. Based on this assumption, we first simulated the genetic association between gene sets and phenotype then simulated the differential expression corresponding to the genetic association.

2.3.1 Gene set data

For each simulation we generated 100 gene sets. Only the first gene set (causal gene set) included risk genes or differentially expressed genes. We randomly chose a pathway, P53PATHWAY that contains 16 genes from MSigDB as a prototype to simulate the causal gene set. The gene expression and genotype information of P53PATHWAY were obtained from breast invasive carcinoma (BRCA) RNA-Seq data and glioblastoma data generated through The Cancer Genome Atlas (TCGA, http://cancergenome.nih.gov) project. The remaining 99 gene sets were simulated from null models, namely none of genes in these gene sets were associated with the phenotype of interest with respect to gene expression profiles or genotypes. The sizes of null gene sets were randomly drawn from U [15, 30]. Genes within null gene sets were randomly drawn from a pool of 984 non-causal genes.

2.3.2 SNP data

Each simulated SNP data set included 1000 genes, each gene with one genotyped SNP for a total of 1000 SNPs. Some of these SNPs were considered causally related to the phenotype of interest. We simulated the causal SNPs in the causal gene set based on the genotype information of P53PATHWAY. We first assigned SNPs that were within the region 1 kilobase pair (kb) upstream of the transcription start site (TSS) to the end of the transcribed bases to each gene in the P53PATHWAY, then we removed SNPs with minor allele frequency (MAF) less than 0.05 and chose the SNP with the highest score in chi-square test as the tag SNP of the gene. We set the allele frequencies of causal SNPs in the simulated causal gene set same as the allele frequencies of corresponding tag SNPs in the P53PATHWAY. The heterozygote odds ratio for each causal SNP was generated from U [1.2, 1.4]. We used an additive disease model for the causal loci and the disease

prevalence was set to 0.05. We drew allele frequencies from a Beta distribution, Beta (0.1, 0.1), for null SNPs with no association with the phenotype based on the approximation for the unconditional distribution of allele frequencies in the HapMap populations stated in (Coram and Tang, 2007)[15]. Based on these parameter settings, the genotype data were generated by PLINK[16] (http://pngu.mgh.harvard.edu/purcell/plink/). We then assigned the case-control status based on the model

$$\text{logit}\{\Pr(Y_j = 1)\} = \sum_{i=1}^{N} x_{G_{ji}} \beta_i + e_j . \qquad (21)$$

Where N is the number of causal SNPs in the causal gene set. $x_{G_{ji}}$ denotes the coding of the genotype at causal SNP i for sample j with effect size β_i that is the log odds ratio at SNP i. e_j denotes a random sample-specific error term for sample j, e_j is sampled from a standard normal distribution.

2.3.3 Gene expression data

Each simulated gene expression data set consisted of 1000 genes corresponding to the 1000 genes in the SNP data set. Some of these genes were considered risk genes that were differentially expressed in cases and controls. We first generated the baseline expression levels for all genes in the data set from the negative binomial (NB) distribution. The NB-distributed read counts were generated by a function in the R Stats Package – rnbinom (n, size, prob, mu). The parameters mu and size were estimated from the normal samples in the TCGA BRCA RNA-Seq data set. Next, we added disease effect to the causal genes in the causal gene set based on the model $x_{ji} = x_0(1 + x_{G_{ji}}\beta)$, where x_{ji} is the expression level of gene i in sample j, x_0 is the baseline expression level of gene i in sample j, $x_{G_{ji}}$ denotes the coding of the genotype at SNP i for sample j in the SNP data. β is the effect size of the genotype on gene expression and reflects the degree to which the gene expression is correlated with the genotype of tag SNP of the same gene. β was drawn from either U [0.8, 1], U[1, 3], or U [2, 4].

References

1. Robinson, M.D., McCarthy, D.J. & Smyth, G.K. edgeR: a Bioconductor package for differential expression analysis of digital gene expression data. *Bioinformatics* **26**, 139-140 (2010).

2. Anders, S. & Huber, W. Differential expression analysis for sequence count data. *Genome Biol* **11**, R106 (2010).
3. Tarazona, S., Garcia-Alcalde, F., Dopazo, J., Ferrer, A. & Conesa, A. Differential expression in RNA-seq: a matter of depth. *Genome Res* **21**, 2213-2223 (2011).
4. Wang, L., Feng, Z., Wang, X., Wang, X. & Zhang, X. DEGseq: an R package for identifying differentially expressed genes from RNA-seq data. *Bioinformatics* **26**, 136-138 (2010).
5. Hardcastle, T.J. & Kelly, K.A. baySeq: empirical Bayesian methods for identifying differential expression in sequence count data. *BMC Bioinformatics* **11**, 422 (2010).
6. Xiong, Q., Ancona, N., Hauser, E.R., Mukherjee, S. & Furey, T.S. Integrating genetic and gene expression evidence into genome-wide association analysis of gene sets. *Genome Res* **22**, 386-397 (2012).
7. Subramanian, A. *et al.* Gene set enrichment analysis: a knowledge-based approach for interpreting genome-wide expression profiles. *Proc Natl Acad Sci U S A* **102**, 15545-15550 (2005).
8. Wang, X. & Cairns, M.J. Gene set enrichment analysis of RNA-Seq data: integrating differential expression and splicing. *BMC Bioinformatics* **14 Suppl 5**, S16 (2013).
9. Wang, X. & Cairns, M.J. SeqGSEA: a Bioconductor package for gene set enrichment analysis of RNA-Seq data integrating differential expression and splicing. *Bioinformatics* (2014).
10. Dinu, I. *et al.* Improving gene set analysis of microarray data by SAM-GS. *BMC Bioinformatics* **8**, 242 (2007).
11. Zaykin, D.V., Zhivotovsky, L.A., Westfall, P.H. & Weir, B.S. Truncated product method for combining P-values. *Genet Epidemiol* **22**, 170-185 (2002).
12. Yu, K. *et al.* Pathway analysis by adaptive combination of P-values. *Genet Epidemiol* **33**, 700-709 (2009).
13. Fu, J. *et al.* Unraveling the regulatory mechanisms underlying tissue-dependent genetic variation of gene expression. *PLoS Genet* **8**, e1002431 (2012).
14. Pickrell, J.K. *et al.* Understanding mechanisms underlying human gene expression variation with RNA sequencing. *Nature* **464**, 768-772 (2010).
15. Coram, M. & Tang, H. Improving Population-Specific Allele Frequency Estimates by Adapting Supplemental Data: An Empirical Bayes Approach. *Ann Appl Stat* **1**, 459-479 (2007).
16. Purcell, S. *et al.* PLINK: a tool set for whole-genome association and population-based linkage analyses. *Am J Hum Genet* **81**, 559-575 (2007).
